# Kawasaki disease vs. MIS-C in a child with congenital coronary artery anomaly: a case report

**DOI:** 10.3389/fped.2026.1768080

**Published:** 2026-02-17

**Authors:** Oksana Boyarchuk, Marta-Viktoriia Zaleshchuk, Roksolana Zaremba, Oksana Chubata, Hanna Morkovkina

**Affiliations:** 1 Department of Children’s Diseases and Pediatric Surgery, Horbachevsky Ternopil National Medical University, Ternopil, Ukraine; 2 Department of Pediatric Nephrology and Cardiorheumatology, Ternopil Regional Children’s Hospital, Ternopil, Ukraine; 3 Department of Radiology, Ukrainian Children's Cardiac Center, Kyiv, Ukraine

**Keywords:** coronary aneurysms, coronary artery anomaly, Kawasaki disease, MIS-C, SARS-CoV-2

## Abstract

Kawasaki disease (KD) and multisystem inflammatory syndrome in children (MIS-C), associated with SARS-CoV-2 infection share overlapping clinical and laboratory features, making differential diagnosis particularly challenging during the COVID-19 pandemic. Accurate distinction is essential due to differences in pathophysiology, management strategies, and cardiovascular outcomes. We report the case of a 7-year-old boy presenting with prolonged fever, mucocutaneous manifestations, arthritis, and elevated inflammatory markers following SARS-CoV-2 exposure. The clinical course demonstrated features compatible with both incomplete Kawasaki disease and MIS-C. Laboratory findings and cardiac biomarkers showed a mixed profile, while echocardiography and coronary imaging revealed the development of coronary artery aneurysms. Notably, a complex congenital coronary artery anomaly was incidentally identified during coronary evaluation. Although such anomalies are not considered independent risk factors for coronary aneurysm formation, their presence may complicate the interpretation of coronary findings in the setting of systemic inflammation. The patient showed a rapid and sustained clinical response to systemic glucocorticoid therapy without intravenous immunoglobulin administration; however, coronary artery aneurysms subsequently developed. This case highlights the diagnostic and therapeutic challenges at the interface of KD and MIS-C and underscores the importance of an integrated, individualized approach that incorporates clinical evolution, laboratory data, and detailed coronary assessment.

## Introduction

Hyperinflammation syndromes represent one of the most challenging clinical entities in pediatric practice, requiring rapid diagnosis and careful differentiation between conditions with overlapping clinical features. Among these, Kawasaki disease (KD) and multisystem inflammatory syndrome in children (MIS-C), associated with SARS-CoV-2 infection occupy a central position, having attracted considerable attention in recent years due to their potentially severe course and high risk of cardiovascular complications (1–3).

KD is an acute systemic vasculitis of medium-sized vessels, with a predilection for the coronary arteries, most commonly affecting children under five years of age. Coronary artery involvement, particularly the development of aneurysms, constitutes the leading cause of long-term cardiovascular morbidity in affected patients (3). In the absence of a specific laboratory biomarker, the diagnosis of KD relies on a combination of clinical presentation and supportive laboratory findings.

Despite substantial advances in understanding KD, its etiology remains incompletely elucidated. Current evidence suggests a multifactorial pathogenesis involving immune dysregulation, infectious triggers, and genetic susceptibility (3–5). Both conventional antigens, which activate a limited fraction of T lymphocytes, and superantigens, capable of stimulating up to 20%–30% of T cells in an MHC-independent manner, have been implicated; these mechanisms may act concurrently and converge on similar pathways of immune activation and vascular inflammation. Support for the role of infectious triggers comes from autopsy studies demonstrating IgA-producing plasma cells within coronary arteries that recognize cytoplasmic inclusion bodies of probable viral origin, as well as increased expression of interferon-response genes in the bronchial epithelium, consistent with responses to respiratory viral infections (4).

During the COVID-19 pandemic, a subset of children developed a hyperinflammatory condition involving multiple organ systems, frequently resembling KD (5, 6). The first reported case of what would later be recognized as MIS-C was described in 2020 in a 9-year-old boy who presented with fever, shock, and multiorgan involvement following SARS-CoV-2 infection (7). By late April 2020, MIS-C was formally defined as a distinct clinical entity based on clusters of similar cases reported in Europe and the United States, characterized by multisystem involvement, cardiovascular dysfunction, markedly elevated inflammatory markers, and temporal association with SARS-CoV-2 infection (8, 9).

MIS-C is currently regarded as a post-infectious inflammatory condition that typically develops 2–6 weeks after SARS-CoV-2 exposure. It is characterized by involvement of two or more organ systems, including cardiovascular, gastrointestinal, hematologic, and mucocutaneous systems along with pronounced elevation of inflammatory markers such as C-reactive protein, D-dimer, ferritin, interleukin-6, and procalcitonin, and a high prevalence of shock and myocardial dysfunction (1, 9, 10).

Despite distinct etiopathogenetic mechanisms, KD and MIS-C share substantial clinical and laboratory overlap, including persistent fever, rash, conjunctivitis, mucosal changes, cardiac involvement, and systemic inflammation (11, 12). This overlap poses significant diagnostic challenges, particularly in cases with incomplete or atypical presentations.

The aim of this report is to present and analyze a clinically challenging case of a 7-year-old boy with overlapping features of Kawasaki disease and MIS-C, illustrating the diagnostic uncertainty that may arise in such presentations. Through a detailed analysis of clinical, laboratory, and cardiologic findings, we compare key characteristics of these two syndromes and discuss the diagnostic and therapeutic challenges encountered in real-world clinical practice. Of particular interest, the disease course was complicated by the development of coronary artery aneurysms in the setting of a congenital coronary artery anomaly.

## Case presentation

A 7-year-old boy was admitted to the pediatric cardiology and rheumatology unit with fever up to 38.5 °C, limping, and severe bilateral hip pain.

The illness began 14 days prior to admission with an acute onset of cervical lymphadenopathy and left-sided neck pain, followed by low-grade fever that progressively increased. Due to parental concern, particularly in light of a previous episode two years earlier characterized by marked lymphadenopathy, fever, and rapid clinical deterioration requiring intensive care, the patient was initially hospitalized in the oncohematology department. The mother also reported recurrent cervical lymph node enlargement during most episodes of acute respiratory viral infections.

At initial admission, physical examination revealed pharyngeal hyperemia and tender posterior cervical lymphadenopathy (up to 3 × 1 cm on the left and 2 × 1 cm on the right), with mobile nodes and intact overlying skin. No other significant abnormalities were identified.

On day 2, body temperature rose to 38.0 °C with mild rhinorrhea. Laboratory evaluation demonstrated leukocytosis (14.76 × 10⁹/L) with neutrophilia (88%), normal platelet count, and elevated C-reactive protein (CRP, 47.2 mg/L) (Supplementary Table S1). A diagnosis of cervical lymphadenitis was made, and antibacterial therapy with adjunctive dexamethasone (0.15 mg/kg) was initiated.

**Figure 1:**
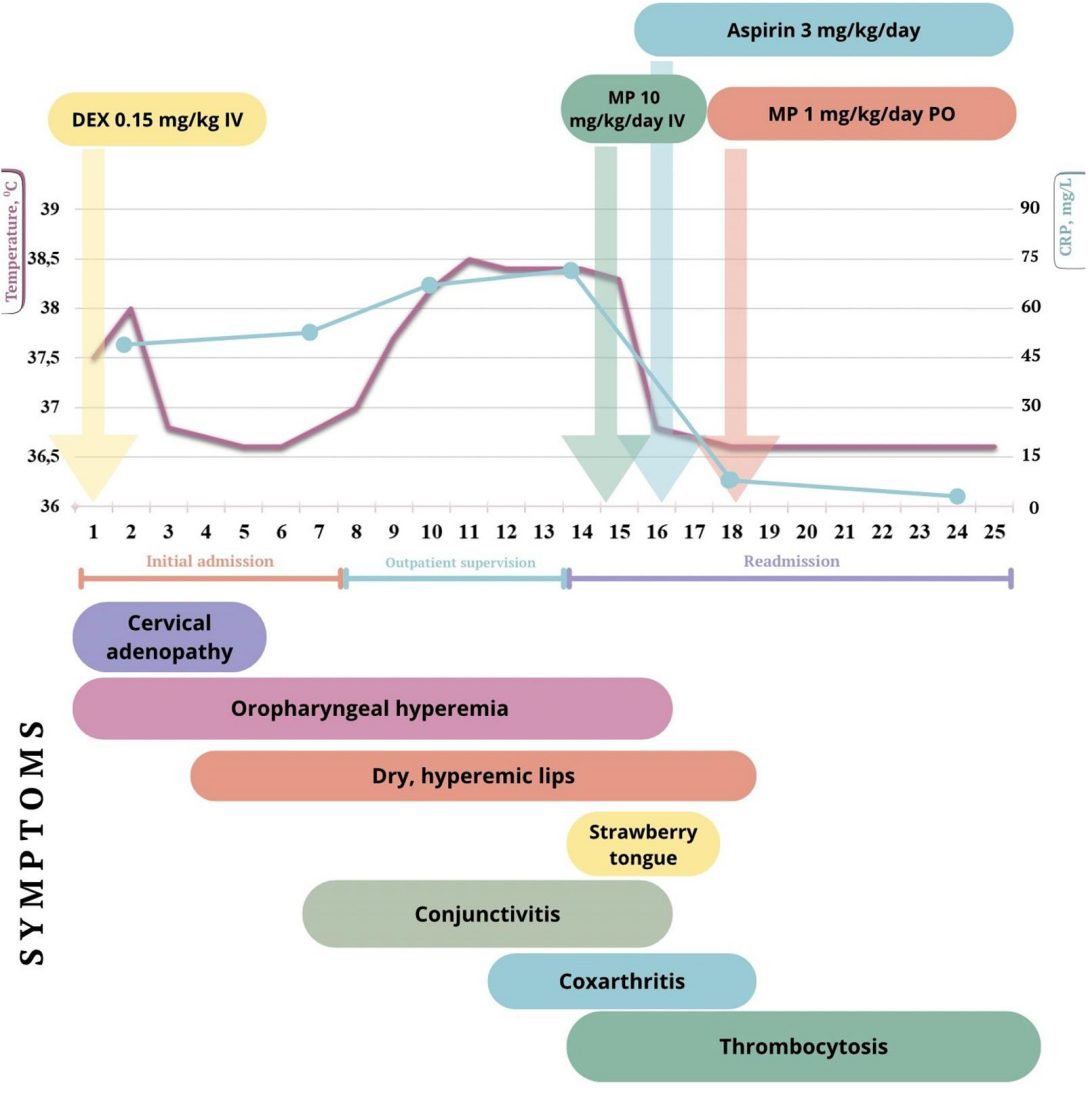
Clinical timeline of disease course, therapeutic interventions, and key clinical manifestations. Temporal relationship between body temperature, CRP, major clinical manifestations, and administered therapies during the course of illness. The figure illustrates the biphasic fever pattern, evolution of mucocutaneous and musculoskeletal symptoms, laboratory thrombocytosis, and the timing of corticosteroid, and antiplatelet therapy. Defervescence occurred rapidly after initiation of high-dose intravenous methylprednisolone (MP), followed by clinical stabilization. DEX, dexamethasone; IV, intravenous; PO, per os.

By day 3, fever resolved and lymphadenopathy regressed; however, lip dryness developed (Figure 1). On day 7 from symptom onset, conjunctival hyperemia appeared, accompanied by persistent cheilitis and mucosal changes. Laboratory tests showed persistent leukocytosis (15.23 × 10⁹/L), elevated ESR (22 mm/h), and CRP of 50.25 mg/L.

**Figure 2:**
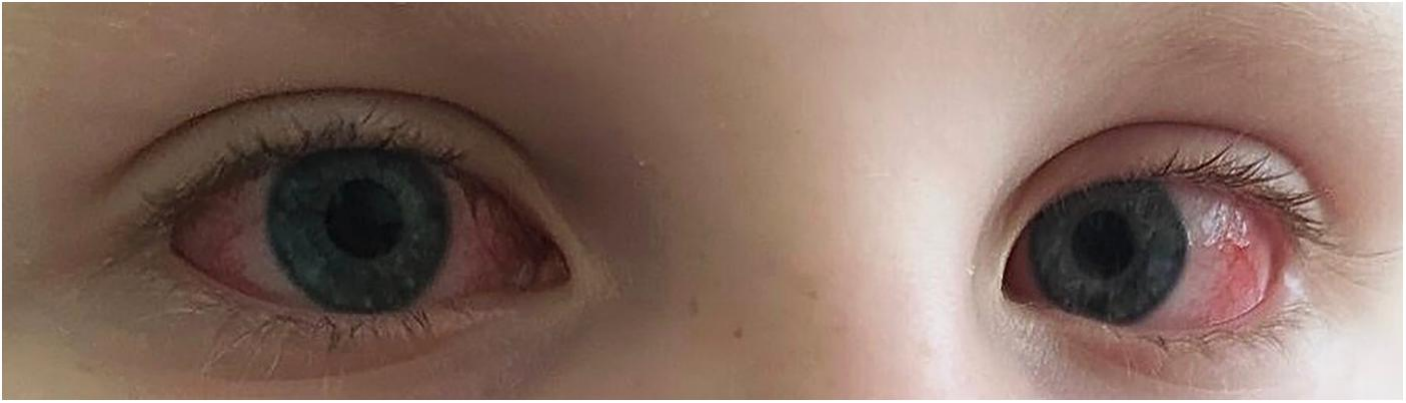
Bilateral non-exudative conjunctival injection with preserved limbal sparing, consistent with dry conjunctivitis observed during the acute phase of the disease.

Subsequently, fever recurred with worsening conjunctivitis (Figure 2). These findings were initially interpreted as adenoviral infection, and the patient was discharged under outpatient supervision. However, clinical deterioration ensued, and on day 12, he developed hip pain and limping, prompting re-admission to the cardiology and rheumatology department.

### Diagnostic assessment

The patient is of Caucasian ethnicity and Ukrainian origin. He was born after an uncomplicated first pregnancy and delivery, with a birth weight of 4,500 g. Vaccinations were administered with deviations from the national schedule. Family history was notable for psoriasis in the paternal grandfather.

At re-admission, anthropometric measurements indicated overweight status (weight 38 kg, z-score +2.15; height 138 cm, z-score +2.10; BMI 20 kg/m^2^, z-score +1.67, 95.3 percentile). Physical examination revealed dry, cracked, and hyperemic lips, strawberry tongue, bilateral non-purulent conjunctivitis, and moderate pharyngeal erythema. Gait was impaired and marked pain during hip joint rotation was observed. Heart rate was 100 beats/min, blood pressure 110/80 mmHg. Cardiac auscultation was normal.

Laboratory findings included persistent leukocytosis, thrombocytosis (459 × 10⁹/L), elevated CRP (71.6 mg/L), and ESR (45 mm/h). Ferritin was moderately increased (375 ng/mL), while lactate dehydrogenase (LDH), aspartate aminotransferase (AST), alanine aminotransferase (ALT), triglycerides, and creatine kinase-MB (CK-MB) were within normal ranges. D-dimer was elevated (1.31 µg FEU/mL, reference <0.5 µg FEU/mL). NT-proBNP was mildly increased (249.6 pg/mL, reference <125 pg/mL), and procalcitonin was 0.09 ng/mL (reference <0.045 ng/mL).

Serology demonstrated high titers of IgG antibodies against the receptor-binding domain of the SARS-CoV-2 spike protein (923.5 AU/mL; 131.1 BAU/mL). Immunophenotyping revealed elevated IgA and IgG levels (Supplementary Table S2).

Electrocardiography showed sinus tachycardia with low QRS voltage. Transthoracic echocardiography revealed preserved left ventricular systolic function (ejection fraction 65%) and no coronary artery dilation at that stage.

Given the prolonged fever, mucocutaneous findings, lymphadenopathy, systemic inflammation, and joint involvement, differential diagnosis included KD, MIS-C associated with SARS-CoV-2, and systemic juvenile idiopathic arthritis (Still's disease) (11, 13). The presence of bilateral conjunctivitis and oral mucosal changes made Still's disease unlikely (14).

### Therapeutic intervention

Based on the diagnostic criteria for KD (3), the patient met three of the five principal criteria in addition to fever—bilateral non-purulent conjunctivitis, oral mucosal changes, and cervical lymphadenopathy, suggestive of incomplete KD. However, considering the patient's age and high titers of SARS-CoV-2 IgG antibodies, MIS-C could not be excluded.

Intravenous methylprednisolone was initiated (10 mg/kg/day for 3 days), followed by oral methylprednisolone (prednisolone equivalent 1 mg/kg/day). Clinical improvement was observed on the second day of steroid therapy, with resolution of fever and significant reduction of joint pain. Low-dose acetylsalicylic acid (100 mg/day; 3 mg/kg/day) was subsequently added (Figure 1).

### Follow-up and outcomes

A follow-up echocardiogram on day 24 from symptom onset showed no coronary abnormalities. Inflammatory markers normalized, and the patient was discharged on day 25 in good clinical condition.

Two weeks later, echocardiography revealed mild coronary dilation (z-score up to +2.5). At 50 days, small coronary artery aneurysms were detected [left main coronary artery (LMCA) z-score +2.46, left anterior descending artery (LAD) + 3.6, right coronary artery (RCA) + 3.62].

**Figure 3:**
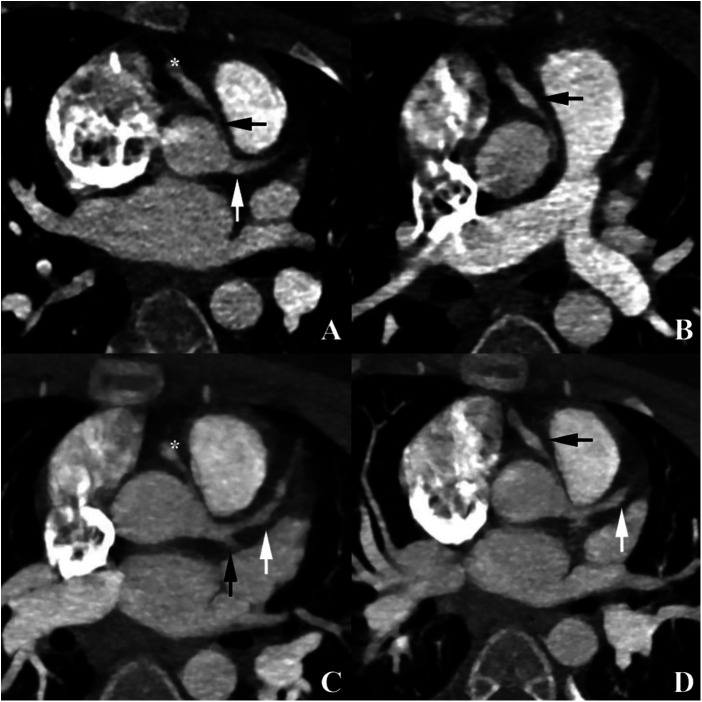
Cardiac computed tomography. Anomalous origin and course of the right coronary artery (RCA) **(A)** Axial image demonstrates an interarterial course of the RCA (black arrow) with proximal compression and aneurysmal dilatation (asterisk). A single coronary artery (white arrow) originates from the left sinus of Valsalva and divides into the left coronary artery and RCA. **(B)** Visualization of aneurysmal dilatation of the RCA (black arrow). **(C)** MIP reconstruction shows pronounced aneurysmal dilatation of the RCA (asterisk) and left anterior descending artery (LAD) (white arrow). The origin of the circumflex artery (Cx) is indicated by a black arrow. **(D)** MIP reconstruction demonstrates aneurysmal dilatation of the RCA (black arrow) and LAD (white arrow).

Cardiac CT performed two months after disease onset demonstrated a single coronary artery originating from the left sinus of Valsalva, with subsequent bifurcation into the right and left coronary arteries (Figure 2), the right coronary artery exhibited an anomalous interarterial course between the aorta and pulmonary artery, with up to 40% luminal compression and aneurysmal dilation of proximal and mid segments which may be consistent with Kawasaki disease–associated coronary arteritis (Figure 3).

At three months, coronary aneurysms persisted without progression. At six months, partial regression of right coronary artery dilation was observed (LCA z-score +2.4; RCA +1.5).

### Patient and family perspective

Following hospital discharge, the patient gradually returned to age-appropriate daily activities without residual systemic symptoms. According to parental report, the child remained clinically well. The family was informed about the nature of the inflammatory condition, the incidental finding of a congenital coronary artery anomaly, and the need for structured long-term cardiology surveillance.

A follow-up plan was established, including serial transthoracic echocardiography at intervals of 1–3 months to monitor coronary artery dimensions and ventricular function. In addition, repeat coronary CT angiography is planned approximately one year after disease onset to reassess coronary anatomy, aneurysm evolution, and the functional significance of the anomalous coronary course. The family expressed understanding of and adherence to the proposed monitoring strategy.

## Discussion

The presented clinical case is distinguished by three key aspects. First, it highlights a common and clinically relevant diagnostic dilemma—differentiation between MIS-C and KD, particularly in the context of ongoing SARS-CoV-2 circulation. Second, it reflects the complexity of therapeutic decision-making in patients diagnosed beyond the early phase of illness, when standard treatment strategies, including intravenous immunoglobulin (IVIG), may require individualization based on clinical course and inflammatory activity. Third, it addresses the potential role of a congenital coronary artery anomaly (CAA) in the pathogenesis and accelerated development of coronary artery aneurysms.

Table 1 summarizes the principal differences between KD and MIS-C associated with SARS-CoV-2. In our patient, several features were consistent with incomplete KD, including unilateral cervical lymphadenopathy, characteristic non-purulent conjunctivitis, absence of prominent gastrointestinal symptoms, development of thrombocytosis from day 13, lack of marked elevation in CRP, absence of severe coagulopathy, and only mild cardiac involvement (borderline NT-proBNP levels with normal CK-MB). Collectively, these findings favored a diagnosis of KD. Conversely, the patient's age, pronounced arthritis, elevated D-dimer and procalcitonin levels, and high titers of anti–SARS-CoV-2 IgG antibodies supported the diagnosis of MIS-C (10, 15). Thus, the clinical presentation represented a mixed or transitional phenotype, complicating diagnostic decision-making.

**Table 1:** Differential diagnosis between kawasaki disease, MIS-C, and patient clinical features.

Criterion/feature	Kawasaki disease	MIS-C	Findings in the patient
Age	Typically <5 years	<21 years	7 years
Clinical criteria			
Fever	≥5 days, usually >38–39 °C	≥24 h, ≥38 °C	Fever ≥38 °C for >5 days
Conjunctivitis	Bilateral, non-exudative; in 90%	Often bilateral, may be exudative; in ∼40%	Bilateral, non-exudative
Oropharyngeal and mucosal changes	Strawberry tongue, dry/cracked red lips, mucosal hyperemia	Lip involvement possible (20%–30%); strawberry tongue less common	Strawberry tongue, dry lips, mucosal hyperemia
Rash	Polymorphous, non-vesicular	Maculopapular, urticarial, vasculitic-like	Not observed
Extremity changes	Edema, erythema; later desquamation	Possible but less common	No
Lymphadenopathy	Unilateral cervical ≥1.5 cm	Often generalized	Unilateral cervical (left-sided)
Cardiac involvement	Possible myocarditis, usually mild; troponin normal or slightly elevated; NT-proBNP often <1,000 pg/mL; systolic function generally preserved (LVEF normal or slightly reduced)	Hypotension in severe cases; shock common; myocarditis/LV dysfunction of varying severity; LVEF often reduced; frequent arrhythmias and conduction disturbances; ↑NT-proBNP, ↑troponin	NT-proBNP 249.6 pg/mL; normal LVEF; low voltage on ECG
Vascular involvement	Untreated it can be associated with coronary artery aneurysms in 20%–25%	Coronary dilatation or small aneurysms in 10%–15%; typically transient (resolve within 1–3 months)	Small and medium coronary aneurysms associated with congenital coronary anomaly
Gastrointestinal involvement	Rare	Very common	None
Joint involvement	Arthralgia/transient arthritis, usually large joints of lower extremities	Often pronounced; multiple joints; pain, impaired movement and gait	Coxarthritis, impaired gait
Laboratory criteria			
CRP	≥30 mg/L (usually up to 100 mg/L)	≥30 mg/L (often markedly elevated, >100–200 mg/L)	Max 70.8 mg/L
ESR	≥40 mm/h	Elevated	Max 45 mm/h
Leukocytes	Leukocytosis ≥15,000/*μ*L with neutrophilia	Often leukopenia or normal WBC	Leukocytosis (15.23 × 10⁹/L), neutrophils 88%
Platelets	Thrombocytosis ≥450,000/μL after day 7–10	Thrombocytopenia common; thrombocytosis not typical	Thrombocytosis 459,000/μL from day 13
Coagulopathy (D-dimer)	Normal or mildly elevated (1,000–2000ng/mL)	D-dimer ↑ (usually markedly high)	1.31 μg FEU/mL (normal <0.5) = 1,310 ng/mL
Procalcitonin	Usually normal	Elevated	0.09 ng/mL (normal <0.046 ng/mL)
Association with SARS-CoV-2	Absent	Detection of viral RNA/antigen or antibodies	IgG antibodies to SARS-CoV-2 positive

AHA, American Heart Association; MIS-C, Multisystem Inflammatory Syndrome in Children; CDC, Centers for Disease Control and Prevention; NT-proBNP, N-terminal pro-B-type natriuretic peptide; LVEF, left ventricular ejection fraction; RCA, right coronary artery; LCA, left coronary artery; LAD, left anterior descending artery; CRP, C-reactive protein; ESR, erythrocyte sedimentation rate; WBC, white blood cells; FEU, fibrinogen equivalent units; RNA, ribonucleic acid.

Previous studies have similarly demonstrated substantial overlap between MIS-C and KD, underscoring the challenges of differential diagnosis based on clinical, laboratory, and cardiovascular features. In a cohort of 277 patients with MIS-C, 45.1% exhibited clinical features overlapping with KD, with incomplete KD accounting for 66.7% of these cases (16).

Age distribution represents an important distinguishing factor. The median age of patients with MIS-C is significantly higher than that of patients with KD (96 vs. 30 months, respectively) (17). In another study comparing 71 KD and 73 MIS-C patients, the mean age was 2.5 vs. 5.7 years (18). The age of our patient (7 years, 92 months) lies within the upper range described for MIS-C and outside the typical age group for classic KD. In addition, MIS-C has been associated with a significantly higher rate of intensive care unit admission compared with KD (34.2% vs. 2.8%) (18).

Fever is a central diagnostic criterion for both conditions. MIS-C is defined by fever ≥38 °C lasting at least 24 h in combination with systemic inflammation (19), whereas KD requires persistent fever for ≥5 days as a cornerstone diagnostic criterion according to American Heart Association guidelines (3).

Regarding other clinical manifestations, gastrointestinal symptoms (nausea, vomiting, abdominal pain) and respiratory involvement (dyspnea) have been reported significantly more frequently in MIS-C (*p* = 0.007, *p* = 0.000, and *p* = 0.002, respectively) (2). Our patient did not exhibit prominent gastrointestinal, a profile more consistent with KD. Furthermore, conjunctivitis (77.5% vs. 68.5%), lymphadenopathy (73.2% vs. 41.1%), and mucosal changes (83.1% vs. 57.5%) have been reported more frequently in KD than in MIS-C (18), further supporting a KD-like phenotype in this case.

Cardiovascular involvement also differs between the two conditions. Coronary artery abnormalities are significantly more common in KD (17), with reported rates of 26.7% in KD vs. 10.9% in MIS-C (18). In contrast, valvular involvement, left ventricular systolic dysfunction, and pericardial effusion occur more frequently in MIS-C (*p* = 0.000, *p* = 0.001, and *p* = 0.023, respectively) (17). Acute cardiac decompensation is a hallmark complication of MIS-C, with approximately one-third of patients demonstrating severely reduced left ventricular ejection fraction (<30%) (9). In our case, no evidence of acute myocardial dysfunction or significant systolic impairment was observed, whereas coronary artery aneurysms developed during follow-up—findings more characteristic of KD.

Immunological profiling further supports a hybrid inflammatory phenotype. Studies comparing MIS-C and KD have demonstrated distinct cytokine signatures, with MIS-C-associated hyperinflammation differing from both KD and acute severe COVID-19. KD is characterized by a Th17-skewed immune response and elevated IL-17A levels, whereas MIS-C generally exhibits lower IL-8 and IL-7 levels, although elevated IL-17A has also been reported in some MIS-C cohorts (20).

A meta-analysis of 12 studies involving 3,073 participants (969 with MIS-C) demonstrated clear laboratory differences between MIS-C and KD. MIS-C was associated with lower leukocyte, lymphocyte, and platelet counts, while neutrophil counts and hemoglobin levels were comparable (21). In our patient, leukocytosis persisted throughout days 2–24 (14.7–19.7 × 10⁹/L), with fluctuating lymphocyte percentages, reflecting a mixed inflammatory pattern. Notably, marked thrombocytosis developed after day 14 (459–611 × 10⁹/L), a hallmark feature of KD, typically peaking in the third week and normalizing within 4–6 weeks, as described by McCrindle et al. (3).

Inflammatory markers such as CRP, D-dimer, and ferritin are generally higher in MIS-C, whereas procalcitonin and ESR show less discriminatory value (21). In this case, elevated CRP (71.6 mg/L), ferritin (375 ng/mL), and D-dimer (1.31 µg FEU/mL) were observed on day 14, alongside mildly increased procalcitonin and ESR, again suggesting a mixed inflammatory profile. Among cardiac biomarkers, only NT-proBNP was mildly elevated, while CK-MB, AST, ALT remained within normal ranges, diverging from the typical MIS-C biochemical profile.

Despite clinical and laboratory overlap, the post-infectious association with SARS-CoV-2 remains a key distinguishing feature of MIS-C. Large cohort studies have shown that most MIS-C patients exhibit positive SARS-CoV-2 serology or other evidence of prior infection, even when PCR testing at admission is negative (10). In contrast, KD has no established causal link to SARS-CoV-2 or any specific infectious agent (3). In our patient, markedly elevated anti–SARS-CoV-2 IgG levels on day 14 support prior infection and argue in favor of MIS-C.

The optimal timing of IVIG administration beyond day 10 of illness remains controversial. While early IVIG administration within the first 10 days is standard for KD, current guidelines (3, 22) allow for later IVIG use in cases of persistent inflammation, fever, or progressive coronary involvement. In this case, IVIG was not administered due to a rapid and sustained clinical response to systemic glucocorticoids, consistent with current MIS-C treatment recommendations, which endorse corticosteroids as first-line therapy or effective monotherapy in selected patients. Large multicenter studies have demonstrated comparable outcomes between glucocorticoid therapy and IVIG, including rates of coronary aneurysm development and resolution (23).

An additional feature of this case was the presence of a complex congenital coronary artery anomaly—a single coronary artery arising from the left sinus of Valsalva with an interarterial course of the right coronary artery and approximately 40% luminal compression. This anomaly was identified incidentally during the evaluation of coronary involvement.

Congenital coronary artery anomalies are occasionally detected during the acute phase of Kawasaki disease and, as reported in previous studies do not appear to independently increase the risk of coronary aneurysm formation (24, 25), Rather, they represent an anatomical variant that may complicate diagnostic interpretation of coronary findings during inflammatory conditions.

In contrast, delayed administration of IVIG is a well-established risk factor for coronary artery aneurysm development in KD. In the present case, the prolonged febrile illness prior to IVIG treatment (>10 days) likely played a central role in the development of coronary dilatations.

The coexistence of an underlying coronary artery anomaly and systemic hyperinflammation added to the diagnostic complexity but should be interpreted with caution. The coronary changes observed in this patient are most plausibly explained by inflammation-related vascular injury associated with KD/MIS-C rather than by the congenital anomaly itself.

This case underscores the importance of early recognition of inflammatory syndromes, timely initiation of IVIG therapy, and careful longitudinal coronary assessment, particularly in patients with atypical anatomy that may confound imaging interpretation.

### Strengths and limitations

This case report has several important strengths. First, it provides a comprehensive and structured comparison between Kawasaki disease and MIS-C in a real-world clinical scenario with overlapping features, integrating clinical presentation, laboratory markers, immunological profiling, and detailed cardiologic assessment. Second, the case is distinguished by the presence of a rare congenital coronary artery anomaly, allowing exploration of the potential interaction between pre-existing coronary anatomy and systemic hyperinflammation in the development of coronary artery aneurysms.

Several limitations should also be acknowledged. As a single-case report, the findings cannot be generalized, and causal relationships between the congenital coronary anomaly, systemic inflammation, and aneurysm formation cannot be definitively established. Immunological analyses were limited to standard lymphocyte subsets and functional assays, without longitudinal cytokine profiling, which could have further clarified the inflammatory phenotype and its evolution over time. In addition, although serological evidence supported prior SARS-CoV-2 infection, the exact timing of infection relative to symptom onset could not be precisely determined. Finally, longer-term follow-up is required to assess the persistence or regression of coronary artery changes and to better understand long-term cardiovascular outcomes in patients with overlapping KD and MIS-C features.

## Conclusions

This case illustrates the diagnostic complexity of differentiating KD from MIS-C during the COVID-19 pandemic, particularly in patients with incomplete or overlapping clinical presentations. The coexistence of clinical features, laboratory abnormalities, and coronary involvement required an integrated, multidisciplinary approach to interpretation and therapeutic decision-making.

A notable aspect of this case was the incidental identification of a complex congenital coronary artery anomaly during the evaluation of coronary involvement. Such anomalies, although not associated with an increased risk of coronary aneurysm formation, may add to diagnostic complexity and require careful interpretation in the context of inflammatory vascular disease.

Overall, the case the case underscores the importance of early recognition of inflammatory conditions, timely initiation of appropriate therapy, careful longitudinal monitoring of coronary arteries, and individualized clinical management, particularly in children with atypical coronary anatomy.

## Data Availability

The datasets presented in this study can be found in online repositories. The names of the repository/repositories and accession number(s) can be found in the article/Supplementary Material.
