# Supplementary material for: Kawasaki disease vs. MIS-C in a child with congenital coronary artery anomaly: a case report
Source: Front Pediatr. 2026 Feb 17;14:1768080. doi: 10.3389/fped.2026.1768080 (PMC12953561; doi:10.3389/fped.2026.1768080)
Supplement: Supplementary file 1 [file Table1.docx]

**Table S1. Clinical data, laboratory test results and treatment of the patient**

| Day of illness | 1 | 2 | 3 | 4 | 5 | 6 | 7 | 8 | 9 | 10 | 11 | 12 | 13 | 14 | 15 | 16 | 17 | 18 | 19 | 24 | 25 |  |
| --- | --- | --- | --- | --- | --- | --- | --- | --- | --- | --- | --- | --- | --- | --- | --- | --- | --- | --- | --- | --- | --- | --- |
| **Dynamics of clinical parameters** | | | | | | | | | | | | | | | | | | | | | | |
| Fever, ^o^C | **37.5** | **38** | N | N | N | N | N | 37 | **37.7** | **38.2** | **38.5** | **38.4** | **38.4** | **38.4** | **38.3** | N | N | N | N | N | N |  |
| Cervical adenopathy | + | + | + | + | ± |  |  |  |  |  |  |  |  |  |  |  |  |  |  |  |  |  |
| Oropharyngeal hyperemia | + | + | + | + | + | + | + | + | ± | ± | ± | ± | ± | + | + | + |  |  |  |  |  |  |
| Dry, hyperemic lips |  |  |  | + | + | + | + | + | + | + | + | + | + | + | + | + | ± | ± |  |  |  |  |
| Strawberry tongue |  |  |  |  |  |  |  |  |  |  |  |  |  | + | + | + | + | - |  |  |  |  |
| Conjunctivitis | - | - |  | - |  |  | + | + | + | + | + | + | + | + | + | + | - | - |  |  |  |  |
| Coxarthritis | - | - |  | - |  |  | - |  | - | - |  | + | + | + | + | + | + | ± |  |  |  |  |
| **Dynamics of laboratory parameters** | | | | | | | | | | | | | | | | | | | | | | **Reference** |
| Leukocytes ,10⁹/L | 9.3 | **14.7** |  |  |  |  | **15.2** |  |  | **13.7** |  |  |  | **15.5** |  |  |  | **15.7** |  | **19.7** |  | 5-11.6 |
| Platelets, 10⁹/L | 168 | 244 |  |  |  |  | 337 |  |  | **396** |  |  |  | **459** |  |  |  | **593** |  | **611** |  | 156-342 |
| ESR, mm/h | 9 | 6 |  |  |  |  | **22** |  |  | **20** |  |  |  | **45** |  |  |  | 10 |  | 4 |  | 4-12 |
| Band neutrophils, % | **7** | **9** |  |  |  |  | 2 |  |  | **12** |  |  |  | **7** |  |  |  | 5 |  | 4 |  | 0.5-5 |
| Segmented neutr, % | 64 | **79** |  |  |  |  | 60 |  |  | **66** |  |  |  | 63 |  |  |  | 60 |  | 45 |  | 35-65 |
| Lymphocytes, % | **20** | **10** |  |  |  |  | 32 |  |  | **15** |  |  |  | **22** |  |  |  | 30 |  | 47 |  | 24-54 |
| Total protein, g/L |  | 75.5 |  |  |  |  | 73.9 |  |  | 72.9 |  |  |  |  |  |  |  | 79.8 |  | 75.2 |  | 60-80 |
| CRP, mg/L |  | **47.2** |  |  |  |  | **50.3** |  |  | **70.8** |  |  |  | **71.6** |  |  |  | **8.22** |  | 0.76 |  | <5 |
| ALT, U/L |  | 19.1 |  |  |  |  |  |  |  | 10.9 |  |  |  | 11.0 |  |  |  | 10.0 |  | 12.2 |  | <41 |
| AST, U/L |  | 21.5 |  |  |  |  |  |  |  | 12.2 |  |  |  | 14.0 |  |  |  | 11.4 |  | 12.2 |  | <50 |
| CK-MB, U/L |  |  |  |  |  |  |  |  |  |  |  |  |  | 12.0 |  |  |  |  |  |  |  | <25 |
| Procalcitonin, ng/mL |  |  |  |  |  |  |  |  |  |  |  |  |  | **0.09** |  |  |  |  |  |  |  | <0.046 |
| Ferritin, µg/L |  |  |  |  |  |  |  |  |  | **268** |  |  |  | **375** |  |  |  |  |  |  |  | 7-140 |
| NT-proBNP, pg/mL |  |  |  |  |  |  |  |  |  |  |  |  |  | **249** |  |  |  |  |  |  |  | < 125 |
| D-dimer, µg FEU/mL |  |  |  |  |  |  |  |  |  |  |  |  |  | **1.31** |  |  |  |  |  |  |  | <0.5 |
| ECG |  |  |  |  |  |  |  |  |  |  |  |  |  |  | **+** |  |  |  |  |  |  |  |
| Echocardiography |  |  |  |  |  |  |  |  |  |  |  |  |  |  | + |  |  |  |  | + |  |  |
| **Treatment** | | | | | | | | | | | | | | | | | | | | | | |
| Corticosteroids | DEX 0.15 mg/kg IV | | | | |  |  |  |  |  |  |  |  |  | MP 10 mg/kg IV | | | MP 1 mg/kg PO | | | |  |
| Antibacterial therapy | Amikacin 250 mg twice daily | | | | | | |  |  |  |  |  |  |  | Ceftazidime 1.0 g twice daily | | | | | | |  |
| Aspirin |  |  |  |  |  |  |  |  |  |  |  |  |  |  |  | 3 mg/kg/day | | | | | |  |

N-normal temperature, ± - partially / mildly present, ESR - erythrocyte sedimentation rate, CRP - C-reactive protein, ALT - alanine aminotransferase, AST - aspartate aminotransferase, CK-MB - creatine kinase–MB fraction, NT-proBNP - N-terminal pro-B-type natriuretic peptide, FEU - fibrinogen equivalent units, ECG – electrocardiography, DEX –dexamethasone, IV – intravenous, MP– methylprednisolone; PO – per os. Hospitalization days are highlighted in blue. The presence of characteristic symptoms is highlighted in pink. Values deviating from the normal range are indicated in bold.
