# Supplementary material for: Kawasaki disease vs. MIS-C in a child with congenital coronary artery anomaly: a case report
Source: Front Pediatr. 2026 Feb 17;14:1768080. doi: 10.3389/fped.2026.1768080 (PMC12953561; doi:10.3389/fped.2026.1768080)
Supplement: Supplementary file 2 [file Table2.docx]

Table S2. Immunologic parameters in presented case

| **Parameter** | **Result** | **Reference range** |
| --- | --- | --- |
| Lymphocytes, cells*/*µL | 3410 | 2000-6000 |
| CD3, % | 79,1 | 57-80 |
| CD3, cells*/*µL | **2697** | 1400-2500 |
| CD4, % | 38,7 | 24-47 |
| CD4, cells*/*µL | 1319 | 700-1500 |
| CD8, % | 24,5 | 19-47 |
| CD8, cells*/*µL | 835 | 600-900 |
| CD19, % | 7,8 | 10-27 |
| CD19, cells*/*µL | 266 | 250-500 |
| Cytotoxic cells (CD3+, CD56+), % | 7,1 | 3-8 |
| NK cells (CD3–, CD56+), % | 6,3 | 4-26 |
| CD14 | 4.2 | 6-13 |
| IgA, g/l | **2.68** | 0,34-2,2 |
| IgM, g/l | 1.35 | 0,4-1,7 |
| IgG, g/l | **15.16** | 5,01-11,7 |
| IgE, IU/ml | 17.7 | <90 |
| Complement C3, g/L | **1.92** | 0,9-1,8 |
| Complement C4, g/L | 0.37 | 0,1-0,4 |
| Lymphocyte proliferative activity | 1,17 | 1,2-1,6 |
| CICs, large, U | 11 | < 20 |
| CICs, medium, U | **116** | 60-90 |
| CICs, small, U | **179** | 130-160 |

Ig – immunoglobulin, IU – international units, CD – cluster of differentiation, NK – natural killer cells, CICs – circulating immune complexes. Values deviating from the normal range are indicated in bold.
